# Adipogenic characterization of immortalized CD55^+^ progenitor cells from human white adipose tissue

**DOI:** 10.1080/21623945.2023.2283213

**Published:** 2023-11-20

**Authors:** Morgane Couchet, Hui Gao, Felix Klingelhuber, Jutta Jalkanen, Thais De Castro Barbosa, Muhmmad Omar-Hmeadi, Lucas Massier, Natalie Krahmer, Niklas Mejhert, Mikael Rydén

**Affiliations:** a Department of Medicine (H7), Karolinska Institutet, Stockholm, Sweden; b Department of Biosciences and Nutrition, Karolinska Institutet, Stockholm, Sweden; c Institute for Diabetes and Obesity, Helmholtz Zentrum München, Neuherberg, Germany. and German Center for Diabetes Research (DZD), Neuherberg, Germany; d German Center for Diabetes Research, Neuherberg, Germany

**Keywords:** Genetic engineering, CRISPR/Cas9, multiomics, spheroid, fat cell, adipocyte, cell model, adipogenesis, lipolysis

## Abstract

**Background:**

Mature adipocytes are difficult to study ex vivo, prompting the use of human adipose progenitor cells (hAPCs). However, hAPCs undergo replicative senescence, limiting their utility in long-term studies.

**Methods:**

We inserted human telomerase reverse transcriptase (TERT) into the AAVS1 locus of CD55+ hAPCs derived from abdominal subcutaneous adipose tissue, and characterized the cells before and after adipogenic differentiation.

**Results:**

TERT-hAPCs retained proliferative and adipogenic capacities for over 80 passages, comparable to early-passage wild type hAPCs. Transcriptomic and proteomic analyses confirmed strong adipocyte gene expression. Functionally, TERT-hAPCs responded to insulin and lipolytic stimuli (isoprenaline, dibutyryl cAMP, TNF-α). They adapted well to both 2D and 3D cultures, with improved adipogenesis under spheroid conditions.

**Conclusion:**

Immortalization of CD55+ hAPCs yields cells with stable proliferative and adipogenic capacity across passages. Being cryopreservable and suitable for both 2D and 3D cultures, TERT-hAPCs offer a reliable, reusable model system for adipocyte studies using cells with a consistent genetic background.

## Introduction

Perturbed white adipocyte function, e.g. alterations in the capacity to store and release lipids, is associated with multiple cardiometabolic complications [1]. To obtain causal insights into these relationships, mechanistic studies of donor-derived fat cells are required. Unfortunately, the buoyancy and fragility of isolated mature fat cells ex vivo limits these analyses. Multiple efforts have therefore been made to create alternative in vitro models [2,3]. Virtually all of these are based on different types of progenitor cells, which are induced to differentiate following the addition of specific pro-adipogenic cocktails. While most of the models are grown in standard 2D cultures, we and others have recently demonstrated that differentiating the cells in organotypic 3D microenvironments results in phenotypes that closely resemble freshly isolated mature adipocytes [4–6].

A common approach to generate adipocytes in vitro is to isolate the stromal vascular fraction (SVF) of white adipose tissue. The SVF contains adipose progenitor cells (hAPCs), which respond readily to adipogenic induction. However, primary hAPCs have limited proliferative capacity and several investigators have therefore immortalized these cells by integrating telomerase reverse transcriptase (TERT) by viral transduction [7–11]. A caveat with this approach is that TERT is randomly introduced into the genome, which may affect the expression of multiple genes and thereby cell function of the edited cells [8,12].

Herein, we immortalized hAPCs from a male donor by knocking-in hTERT in a safe harbour locus by Cas9-mediated engineering. Our characterizations, which include functional and transcriptomic/proteomic profiling, demonstrate that the edited cells retain adipogenic capacity and hormonal sensitivity/responsiveness without signs of replicative senescence for >80 passages in both 2D and 3D culture conditions.

## Results

### Generation of immortalized hAPCs

**Figure 1.**
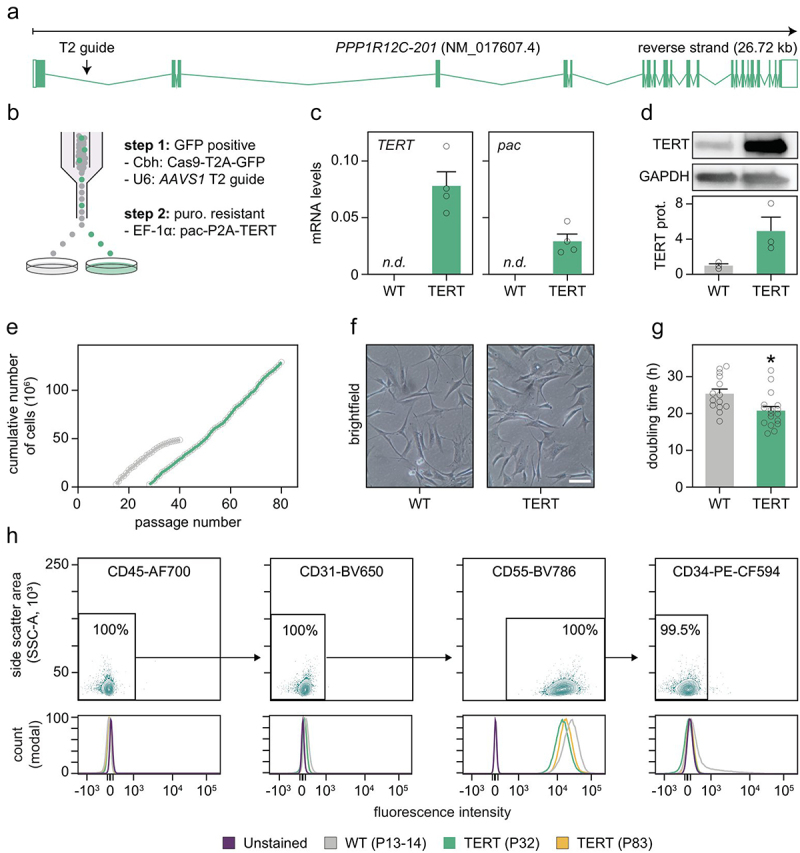
Targeted knock in of TERT-hAPCs results in cells with improved proliferative capacity.(a) Overview of the AAVS1 safe harbor *PPP1R12C* locus, including the T2 guide targeting site. (b) Strategy for establishing TERT-hAPCs using dual selection steps. (c) Messenger RNA levels of *TERT* and *pac* gene in both WT- and TERT-hApcs (*n* = 4). n.d.=not detectable. (d) TERT protein quantification in WT- and TERT-hApcs (*n* = 3). (e) Cumulative numbers of WT- and TERT-hAPCs over multiple passages. (f) Representative images of WT- (P19) and TERT-APCs (P65) in the proliferative state. Scale bar is 100 μm. (g) Comparison of doubling times between WT- (25.4 h, *n* = 14) and TERT-hAPCs (20.8 h, *n* = 17). Statistical differences were determined by Mann–Whitney U test. (h) Quantification of selected cell surface markers in WT- and TERT-hAPCs in the indicated passages by flow cytometry (data from one representative run are shown).

To immortalize hAPCs, we knocked in the EF-1 alpha promoter driving the expression of Puromycin N-acetyltransferase-P2A-hTERT in the *AAVS1* safe harbour locus (also known as *PPP1R12C*) by Cas9-mediated engineering (Figure 1(a)). For this, we electroporated a repair template together with a bicistronic vector expressing the AAVS1-T2 targeting guide RNA as well as Cas9-T2A-EGFP. GFP^+^ cells were selected using fluorescence-activated cell sorting (FACS) and subsequently cultured in puromycin-containing media (Figure 1(b)). We opted to use double selection to increase the proportion of edited cells. To confirm successful genome integration, we analysed the mRNA expression of *TERT* and the gene encoding Puromycin N-acetyltransferase (*pac*) in edited and non-edited hAPCs. Our results showed qPCR-detectable expression of these two genes only in edited cells (Figure 1(c)). These findings were confirmed by western blot, where TERT protein expression was barely detectable in non-edited, wild-type (WT) cells compared to immortalized hAPCs (Figure 1(d)). We named these immortalized cells TERT-hAPCs and refer to non-edited cells henceforth as WT-hAPCs.

We next determined the proliferative capacity of TERT-hAPCs and WT-hAPCs by counting the cumulative number of cells over multiple passages (Figure 1(e)). As expected, WT-hAPCs, counted from passage 15 to passage 40, showed signs of replicative senescence after approximately 30–35 passages. In contrast, TERT-hAPCs displayed a consistent increase in cell number between passage 28 to 80 with no obvious change in cell morphology over time compared to WT-hAPCs (Figure 1(f)). These assessments were complemented by quantifying doubling times which were significantly lower in TERT-hAPCs (on average 20.8 h for passage 31–81) compared to WT-hAPCs (on average 25.4 h for passage 12 to 31) (Figure 1(g)).

### TERT-hAPCs remain CD55^+^ across multiple passages

Both human and murine APCs constitute a heterogeneous cell population where the subset marked by CD55 is capable of differentiating into committed preadipocytes [13,14]. This is corroborated by a recent meta-analysis of single-cell data, wherein CD55^+^ cells were described as primordial adipocyte progenitors in human white adipose tissue [15]. To test which type of hAPC the non-edited and edited cells represented and whether prolonged in vitro proliferation or gene editing influenced this, we performed FACS on WT-hAPCs (P13) as well as TERT-hAPCs at early (P32) and late (P83) passages (Figure 1(h)). As expected, our analyses showed that all cells were negative for leukocyte (CD45) and endothelial (CD31) cell markers. In contrast, cells were clearly positive for CD55 and remained so across passages. In concordance with previous results, the CD55^+^ cells were negative for CD34, which is a cell stemness marker downregulated by *in vitro* culture [16,17].

### TERT-hAPCs retain the capacity to differentiate into adipocytes over multiple passages

**Figure 2.**
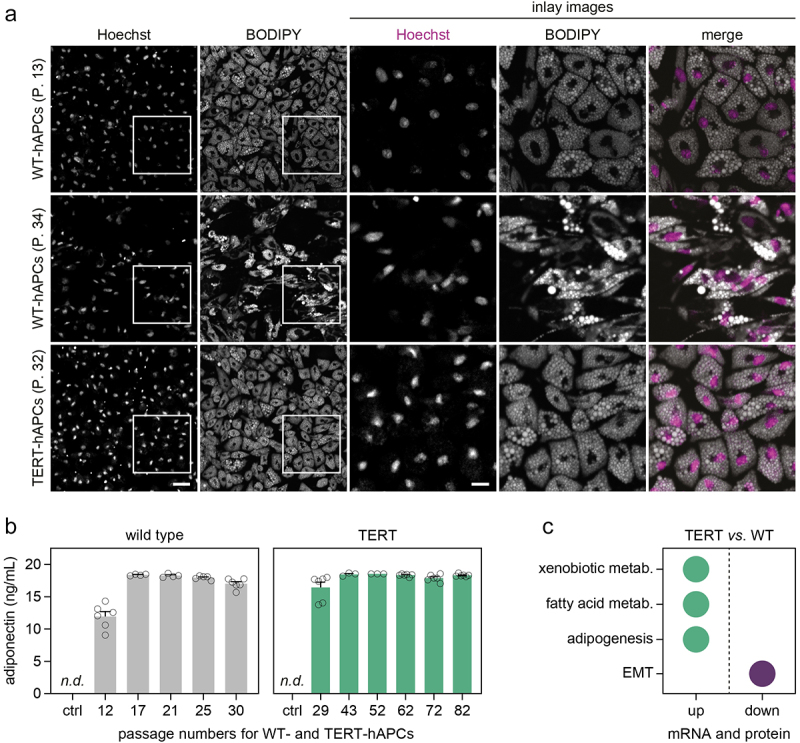
WT- and TERT-hAPCs display high adipogenic capacities.(a) Representative images of WT- and TERT-hAPCs at the indicated passages. Scale bars, 50 μm and 20 μm (inlay). (b) Adiponectin levels in conditioned media in undifferentiated (ctrl) and differentiated of WT- and TERT-hAPCs. (c) Pathway analysis of genes/proteins up- or down-regulated in TERT- *vs*. WT-hAPCs. EMT= epithelial-to-mesenchymal transition.

We tested the adipogenic capacity of TERT-hAPCs in standard 2D culture following induction with a pro-adipogenic cocktail. Results were compared with WT-hAPCs for up to 30–35 passages as the unedited cells underwent replicative senescence after that. As displayed in Figure 2(a,b), we determined lipid accumulation and adiponectin secretion at the end of adipogenesis across passages. While TERT-hAPCs retained a similar degree of lipid accumulation over time, WT-hAPCs displayed fewer cells with heterogeneous lipid accumulation at later passages, a phenotype which was also observed in two other available human adipocyte models (hWA [7] and ASC52telo [11], Figures 2(a) and S1A-C). Furthermore, both cell types secreted similar amounts of adiponectin and in TERT-hAPCs, these levels were unaltered up to >80 passages (Figure 2(b)). To further characterize the cells, we performed RNA-sequencing and proteomic analyses of TERT-hAPCs and WT-hAPCs as described in the Methods section. Data integration of the transcriptome and proteome profiles allowed us to identify 4700 genes, which were also detected at the protein level by mass-spectrometry. Out of these, 588 entries displayed congruent mRNA and protein regulation (402 higher and 186 lower, false discovery rate < 0.05) comparing edited vs. non-edited adipocytes. The significantly altered subsets were used as inputs to identify pathways that differed between the two hAPCs. Our results revealed that ‘adipogenesis’ (e.g. *ACOX1*, *ALDH2*, *CD36*), ‘fatty acid metabolism’ (e.g. *CPT2*, *GPD2*, *MGLL*) and ‘xenobiotic metabolism’ (e.g. *AKR1C2*, *AKR1C3*, *CBR1*) were enriched in TERT-hAPCs *vs*. WT-hAPCs (Figure 2(c), individual genes/proteins listed in Tables S1–2). In contrast, ‘epithelial-mesenchymal transition’ represented by several genes encoding collagens and extracellular matrix (ECM) proteins (e.g. *COL1A1*, *COL3A1*, *COL6A3*) was lower in TERT-hAPCs (Figure 2(c)). Altogether, this shows that TERT-hAPCs retain full capacity to differentiate into adipocytes over multiple passages with limited impact on most cellular pathways.

### TERT-hAPCs differentiate into functional adipocytes

**Figure 3.**
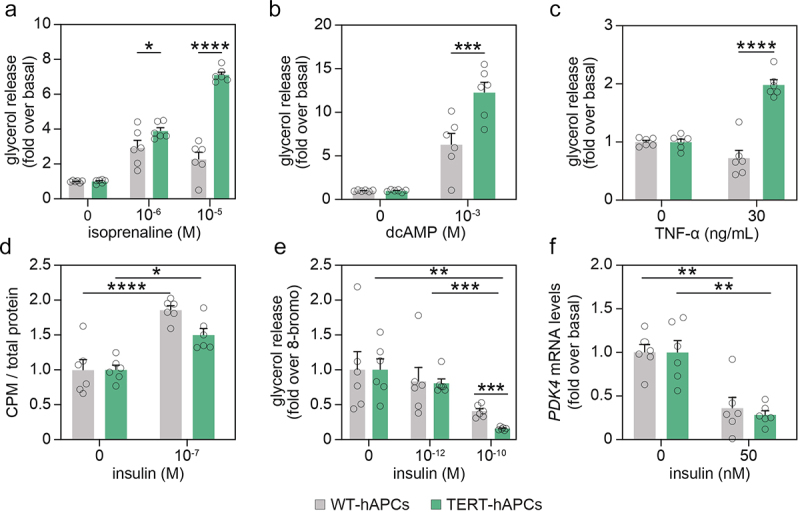
TERT-hAPCs display lipid handling characteristics similar to WT-APCs. (a-c) Lipolysis induced for three hours by the non-selective beta-adrenergic receptor agonist isoprenaline (a), the phosphodiesterase-resistant cAMP analogue dcAMP (b) and TNF-α (c). Statistical differences were determined by two-way ANOVA followed by a Sidak post-hoc test. (d) Basal and insulin-stimulated lipogenesis was quantified in WT- and TERT-hAPCs. In panels A-D, WT-APCs were used at passage 15 and TERT-hAPCs at passage 50. (e) Glycerol release following incubation without or with insulin at indicated concentrations expressed as fold-change over 8-bromo cAMP-stimulated lipolysis. (f) *PDK4* mRNA levels determined by qPCR in WT-APCs (passage 15) and TERT-hAPCs (passage 32) incubated without or with insulin at the indicated concentration. Statistical differences were determined by two-way ANOVA followed by a Sidak post-hoc test. *= *P*<0.05, **= *P*<0.01, ***= *P*<0.001, ****=*P*<0.0001.

We followed-up on this mapping by concentrating our studies on two hallmark functional features of differentiated adipocytes, namely lipolysis and lipogenesis. Short-term incubation with the beta-adrenergic receptor agonist isoprenaline and the phosphodiesterase-resistant cAMP-analogue dibutyryl cAMP (dcAMP) resulted in a pronounced increase in glycerol release from both edited and non-edited adipocytes (Figure 3(a,b)). In addition to these two compounds, we also tested the effect of the cytokine TNF-α which has been shown to induce lipolysis in primary human fat cells [18]. While TNF-α did not increase glycerol release in WT-hAPCs, we observed a two-fold induction in TERT-hAPCs (Figure 3(c)), which is comparable to the effects observed in primary *in vitro* differentiated adipocytes [18]. To measure the responsiveness to insulin, we determined *de novo* lipogenesis in the absence/presence of insulin. This showed increased glucose incorporation into lipids upon insulin stimulation in both cell types (Figure 3(d)). To complement these investigations, we also tested the effects of insulin on lipolysis (concentration-dependent) and gene expression. Our results showed that TERT-hAPCs retained similar degrees of insulin sensitivity at early and late passages (Figures 3(e,f) and S2A-B). Taken together, following *in vitro* differentiation, TERT-hAPCs display functional hallmarks of mature white adipocytes.

### TERT-hAPCs form adipocyte spheroids

**Figure 4.**
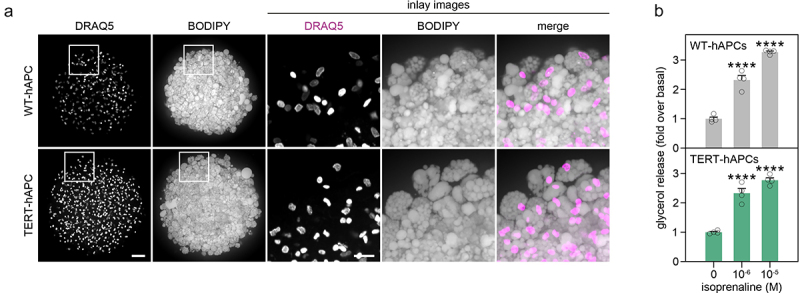
TERT-APCs differentiate into adipocytes in 3D cultures. (a) Representative images of WT- and TERT-hAPC spheroids. Results are presented as maximum intensity projections from Z-stacks of images. Scale bars, 50 μm and 20 (inlay). (b) both WT- and TERT-hAPCs 3D-spheoroids respond to isoprenaline-stimulated lipolysis (*n* = 4). Statistical differences were determined by one-way ANOVA followed by a Tukey’s post-hoc test. WT- and TERT-hAPCs were used at passage 15 and 50, respectively. ****=*P*<0.0001.

Finally, we tested the ability of TERT-hAPCs and WT-hAPCs to form spheroids by plating undifferentiated cells in ultra-low-attachment plates. Following adipogenic induction, both cells were able to differentiate into adipocyte spheroids with paucilocular lipid droplets (Figure 4(a)). We next tested the ability of isoprenaline to hydrolyse triacylglycerols and observed that both cell types responded with an increased glycerol release (Figure 4(b)). We complemented these studies by comparing the proteomic profiles of TERT-hAPCs and WT-hAPCs differentiated in 2D and 3D conditions, respectively (Tables S3–4). This revealed that in both cell types, spheroid cultures compared with standard 2D conditions were enriched for proteins involved in ‘adipogenesis’ (e.g. ADIPOQ, FABP4, LPL), ‘oxidative phosphorylation’ (e.g. DLAT, PDHB), ‘protein secretion’ (e.g. IGF2R, ABCA1, CD63), ‘peroxisome’ (e.g. ABCD2, MLYCD), and ‘cholesterol homeostasis’ (e.g. EBP, FDPS, SCSD) (Table S5). Thus, our results align with recent data demonstrating that culturing cells in 3D results in profiles that differ extensively from the classical 2D models [4]. In contrast, comparisons of TERT-hAPCs and WT-hAPCs in 3D showed only minor differences. In fact, the only pathway that differed between the two hAPCs was ‘epithelial mesenchymal transition’ (e.g. COL6A2, COL1A1, COL6A3), where several collagens were higher in TERT-hAPCs (Table S6). Altogether, our data show that TERT-hAPCs can be used in both adipocyte 2D and 3D cultures.

## Discussion

In this report, we describe the generation of immortalized CD55^+^ hAPCs with pronounced proliferative and adipogenic capacities. By characterizing these cells, we show that they display protein secretion, lipid content and turnover as well as transcriptional and proteomic fingerprints of adipocytes following in vitro differentiation in both 2D and 3D cultures. Thus, the knock-in strategy described herein allows the establishment of an expandable cell source for CD55^+^ hAPCs, which enables advanced in vitro studies in adipocytes derived from the same genetic background.

As extensively discussed elsewhere [19], there are several methods to immortalize cells. One common way is by stably expressing TERT using viral transduction and subsequent cell selection. This approach results in random integration, which together with potential effects of viral transduction, may influence cell function. For SVF-derived hAPCs, random TERT-integration has indeed been reported to impair adipocyte differentiation and function [8,12]. In this study, we therefore capitalized on a targeted system where we integrated TERT into the *AAVS1* safe harbour locus, which has been shown to allow gene integration with limited off-target effects [20,21]. In line with this, we demonstrate that the edited cells display a similar phenotype compared with non-edited cells without signs of replicative senescence. Therefore, our protocol for Cas9-mediated engineering of hAPCs, provides multiple advantages compared with prior approaches.

Our study is based on human CD55^+^ cells from the abdominal subcutaneous region. Cells marked by this surface protein have been shown to reside in many different tissues and have the capacity to differentiate into multiple lineages, including adipocytes [22]. This is, however, not the only progenitor subtype present in adipose tissue as recent single-cell studies based on adipose SVF have shown that several APCs exist. These include two anti-adipogenic populations termed fibroinflammatory progenitors [23,24] and adipogenesis-regulatory cells [13,14]. Whether it is possible to isolate and immortalize these and other types of APCs resident in different WAT depots is presently not known. However, if feasible, this could potentially open avenues for more advanced co-culture studies, which could improve our understanding of the interplay between different APCs and how they contribute to tissue function.

Edited and non-edited cells were analysed by transcriptomic and proteomic profiling. This multi-omic approach allowed us to identify consistent differences between hAPCs at the mRNA and protein levels. In line with the functional analyses, we found that under both 2D and 3D culture conditions, TERT-hAPCs were similar to WT-hAPCs. Admittedly, TERT-hAPCs displayed lower levels of a limited set of ECM proteins under 2D conditions, which were higher under 3D conditions compared with non-edited cells (e.g. COL1A1, COL6A3, TGFB1, VCAN). We interpret these as minor stochastic differences with limited effects on adipocyte function.

Translational advances in metabolic research requires different human cell models. Our approach shows that CD55^+^ hAPCs can be efficiently immortalized with limited effects on gene/protein expression and adipogenic capacity. However, the impact of introducing the TERT sequence, even in a safe harbour, remains to be fully explored and prolonged in vitro culture may induce genetic drift. Thus, the immortalization protocol may result in differences that we have not explored in the present work. Nevertheless, this model can be a valuable addition to current cell systems in the field of adipocyte research.

## Materials and methods

### Isolation and immortalization of WT-hAPCs

Subcutaneous hAPCs were isolated from a liposuction obtained from a male with a BMI of <25 kg/m^2^. Ethical approval for studies of adipose tissue collected in connection with liposuctions was obtained from the regional ethics board in Stockholm. The donor was anonymous to all involved researchers and informed written consent to donate tissue was provided to the surgeon who was not involved in the immortalization and characterization of the cells. A total of 1.4 L lipoaspirate was rinsed with PBS in a large strainer and incubated with collagenase from *Clostridium histolyticum* (#C0130-5 G, Sigma-Aldrich) for 40 min at 37°C. The mixture was shaken every 10 min and following digestion filtered through 200 µm nylon strainers. The floating adipocytes were thereafter discarded and the stromal vascular infranatant collected and spun at 200 × *g* for 10 min. The resulting cell pellet containing 23.2 million viable cells was collected and plated in proliferation media (DMEM low glucose [#31885–023, Thermo Fisher Scientific] supplemented with 10% FBS [#SV30160, Hyclone], 10 mM HEPES [#15630–056, Thermo Fisher Scientific], 50 I.U./mL penicillin and 50 μg/mL streptomycin [#15140122, Thermo Fisher Scientific]). Cells were washed twice after 14 h to remove debris and slowly adhering cells. To immortalize the cells, the EF-1 alpha promoter driving the expression of Puromycin N-acetyltransferase-P2A-TERT was stably integrated in the adeno-associated virus integration site 1 (*AAVS1*) locus (also known as the *PPP1R12C* locus). The Neon transfection system was used to electroporate cells with 1 µg of each plasmid per 1 million cells, settings: 1150 V, 30 ms, 2 pulses, Thermo Fisher Scientific. The following two plasmids were used: **i)** pSpCas9(BB)-2A-GFP [#48138, Addgene], in which the AAVS1-T2 targeting guide RNA sequence (5’-GGG GCC ACT AGG GAC AGG AT-3’) was ligated in using the *option b* protocol described in Ran *et al.* [25] and **ii)** pSH-EFIRES-P-AtAFB2 [#129715, Addgene], which was digested using BglII [#R0144, NEB] and NotI [#R0189, NEB] and thereafter ligated with Puromycin N-acetyltransferase-P2A-TERT. For the insert, TERT was PCR amplified from the pBABE-puro-hTERT plasmid [#1771, Addgene] and Puromycin N-acetyltransferase-P2A sequences were ordered as a gBlock Gene Fragment (Integrated DNA Technologies). The digested pSH-EFIRES-P-AtAFB2 backbone was isolated by gel electrophoresis, followed by gel extraction of the DNA using NucleoSpin Gel and PCR Clean-up [#740609, Macherey-Nagel], ligated using T4 DNA ligase [#M0202, NEB] and transformed into One Shot™ Stbl3™ Chemically Competent cells [#C737303, Thermo Fisher Scientific]. Two days following electroporation, GFP^+^ cells were selected using BD FACSAria Fusion (BD Biosciences) and expanded. Subsequently, a second selection step was performed by incubating the cells with puromycin [2 µg/mL, #A1113803, Thermo Fisher Scientific] during three to four days. The media containing puromycin was changed every day, and the selection was stopped when unedited control cells cultured in parallel wells were no longer viable.

### Cell proliferation and differentiation of WT- and TERT-hAPCs

Cells were cultured at 37°C in a humidified atmosphere with 5% CO_2_ in proliferation media (described above) supplemented with 2.5 ng/mL fibroblast growth factor 2 (FGF2), [#F0291, Merck]. Three days after confluence, FGF2 was removed from the media (day −1) and differentiation was induced the next day (day 0) using differentiation media composed of William’s E medium [#A1217601, Thermo Fisher Scientific] supplemented with 2 mM L-glutamine [#25030024, Thermo Fisher Scientific], 50 I.U./mL penicillin and 50 μg/mL streptomycin [#15140122, Thermo Fisher Scientific], 1× insulin (10 µg/mL), transferrin (5.5 µg/mL, selenium solution (0.0067 µg/mL) [#4140045, Thermo Fisher Scientific], 100 nM dexamethasone [#D1756, Merck], 10 µg/mL transferrin [#T8158, Merck], 500 μM 3-Isobutyl-1-methylxanthine [#I5879, Sigma-Aldrich], 10 nM cortisol [#H0396, Sigma-Aldrich], 2 nM 3,3*'*,5-Triiodo-L-thyronine [#T6397, Sigma-Aldrich], 10 µM rosiglitazone [#71740, Cayman Chemical], 33 µM biotin [#B4639, Sigma-Aldrich], and 17 µM pantothenic acid [#P5155, Sigma-Aldrich]. Proliferation and differentiation media were replaced every three to four days until adipocytes were fully differentiated (2D culture: day 13, 3D culture: day 17). For spheroid formation, cells were cultured in 96 wells ultra-low attachment round bottom plates [#CLS7007, Merck] as described before [4]. In brief, spheroids were differentiated with all the components present in the differentiation media until day 17, followed by six days in William’s E medium supplemented with L-glutamine, penicillin/streptomycin, 1× insulin, transferrin, selenium solution and dexamethasone. To evaluate insulin’s effects on gene expression and lipolysis, the insulin concentration on differentiation day 10 was reduced 20-fold (0.25 µg/mL) and completely omitted on differentiation day 12. On the day of the experiment (day 13), the media was changed to fresh medium without insulin for 3 h. Cells were then treated without or with insulin [#I9278, Sigma-Aldrich] for 2 or 3 h for analyses of gene expression or lipolysis, respectively.

### TERT-hWA and ASC52telo cultures

TERT-hWA were proliferated in Advanced DMEM/F12 [#12-634-010, Thermo Fisher Scientific] supplemented with 10% FBS, L-glutamine (2 mM), penicillin (62.5 μg/ml), streptomycin (100 μg/ml), and basic fibroblast growth factor (bFGF) (2.5 ng/ml) as previously described [7]. ASC52telo (ATCC® SCRC-4000™) were proliferated in DMEM low glucose supplemented with 10% FBS, 50 I.U./mL penicillin and 50 μg/mL streptomycin as described [11]. Both cell types were differentiated in supplemented William’s E medium as described above up to day 13.

### Proliferation capacity and doubling times

To assess proliferation and doubling times, WT-hAPCs and TERT-hAPCs were trypsinized and equal numbers of cells were plated in proliferation media. After 12, 24, 36, 48, 72 h of incubation, cells were collected and counted using Bürker counting chambers. Doubling times were calculated as described previously [7] using the following formula:

$$\mathrm{Doubling}\;\;\mathrm{time}\left(\mathrm{days}\right)\;\;\;\;\;\;\;\;\;\;\;\;\;\;\;\;\;\;\;\;\;\;\;\;\;\;\;\;\;\;\;\;\;\;\;\;\;\;\;\;\;\;\;\;\;\;\;\;\;\;\;\;\;\;\;\;\;\;\;\;\;\;\;\;\;\;\;\;\;\;\;\;\;\;\;\;\;\;\;\;\;\;\;\;\;\;\;\;\;\;\;\;\;\;\;\;\;\;\;\;\;\;\;\;\;\;\;\;=\frac{\mathrm{Duration}\left(\mathrm{days}\right)*LOG\left(2\right)}{\left(\mathrm{LOG}\left(\mathrm{Number}\;\;\mathrm{of}\;\;\mathrm{cells}\;\;\mathrm{final}\right)\right)-\left(\mathrm{LOG}\left(\mathrm{Number}\;\;\mathrm{of}\;\;\mathrm{cells}\;\;\mathrm{initial}\right)\right)}$$

### Flow cytometric analysis

To quantify the abundance of selected surface markers, cells were dissociated with trypsin, pelleted (200 × *g* for 10 min), and resuspended in washing buffer (PBS supplemented with 0.5% BSA [#A4503, Sigma-Aldrich] and 2 mM EDTA [#E7889, Sigma-Aldrich]). Subsequently, the cells were passed through a 35 µm cell strainer [#7340001, VWR], centrifuged as above, and resuspended in antibody solution containing the following fluorophore-labelled antibodies: the pan-leukocyte marker CD45-Alexa Fluor 700 [1:100, #560566, BD Biosciences], the endothelial marker CD31-Brilliant Violet 650 [1:100, #740571, BD Biosciences], as well as the progenitor markers CD34-PE-CF594 [1:100, #562449, BD Biosciences] and CD55-Brilliant Violet 786 [1:40, #742681, BD Biosciences]. Samples were incubated in dark at 4°C for 30 min washed, resuspended in FACS buffer (PBS supplemented with 0.1% BSA and 2 mM EDTA) and filtered through a 35 µm cell strainer. Dead cells were excluded using the 7AAD-dye [1:10 000, #559925, BD Biosciences], which was added to the samples 10-min prior to analysis. BD LSRFortessa flow cytometer equipped with 355, 405, 488, 561 and 640 nm lasers, and DIVA software (BD Biosciences) was used to record 100,000 events per sample. Live gate, light scatter gate, doublet discrimination gate, and fluorescent gates CD45, CD31, CD55 and CD34 set according to fluorescence minus one – controls were applied and the data analysis was performed with FlowJo Software v10.8.0 (BD Biosciences).

### RNA isolation, cDNA synthesis and real-time qPCR

Total RNA was purified using the NucleoSpin RNA kit [#740955, Macherey-Nagel] and the concentration and purity of the samples were measured using a NanoDrop 2000 spectrophotometer [Thermo Fisher Scientific]. Reverse transcription and mRNA levels were performed/analysed with iScript cDNA synthesis [#1708891, BioRad] and iQ SYBR® Green Supermix [#1708882, BioRad] kits, respectively. Relative expression levels were calculated with the comparative Ct-method: 2^ΔCt-^^target^ ^gene^/2^ΔCt-^^reference^ ^gene^. The following primers were used: *TERT* (FW: 5’-GAG AAC AAG CTG TTT GCG GG-3’; RV: 5’-AAG TTC ACC ACG CAG CCA TA-3’), *pac* (FW: 5’-GTC TGG GTA GTG CCG TAG TG-3’; RV: 5’-ATT CCG TGG TGC GCT AGT TT-3’), *B2M* (FW: 5’-AAG GAC TGG TCT TTC TAT CTC-3’; RV: 5’-GAT CCC ACT TAA CTA TCT TGG-3’), *PDK4* (FW: 5’- CTA CTG GAC TTT GGT TCA GAA A-3’; RV: 5’- GCA CTG AAG AGG TAT TTA CTA ATT G-3’) and *18S* (FW: 5’-TGA CTC AAC ACG GGA AAC C-3’; RV: 5’-TCG CTC CAC CAA CTA AGA AC-3’).

### Western blot analyses

Cells were lysed in RIPA buffer [#89901, Thermo Fisher Scientific] supplemented with 1× protease inhibitor cocktail [#11836170001, Merck] as well as 1× phosphatase inhibitors [#4906837001, Merck]. Protein concentrations were determined using the Pierce BCA kit [#655101, Greiner-bio One]. 20 µg aliquots of total protein were mixed with Laemmli buffer and heated at 95°C for 5 min. Proteins were separated by SDS – PAGE and transferred to PVDF membranes [Transblot turbo kit #1704274, BioRad]. Membranes were incubated in blocking solution (3% of ECL blocking agent [#RPN418, GE Healthcare] in Tris-buffered saline supplemented with 0.1% Tween 20 (TBST)) for 1 h at room temperature and subsequently in blocking solution supplemented with primary antibody (anti-telomerase reverse transcriptase [1:1000, #ab32020, Abcam] and anti-GAPDH [1:1000, #2118, Cell Signaling]) overnight at 4°C. After several washes in TBST, membranes were incubated in blocking solution supplemented with horseradish peroxidase-conjugated mouse anti-rabbit secondary antibody [1:10000, #7074S, Cell Signaling] for 1 h at room temperature, washed several times in TBST, and incubated with ECL western-blotting detection reagent [#12644055, Thermo Fisher Scientific]. Images were acquired using the ChemiDoc MP Imaging System (Bio-Rad Laboratories).

### Adiponectin secretion

Conditional media from cells, before and after differentiation, were collected 3 days after the last media change to determine adiponectin secretion by ELISA following the protocol provided by the manufacturer [#DRP300, R&D systems].

### Basal and stimulated lipolysis

Cells were incubated in lipolysis medium (phenol red-free DMEM/F12 [#7074S, Gibco] supplemented with 2% BSA [#A4503, Sigma-Aldrich]) without (basal) or with isoprenaline [#I5627, Sigma-Aldrich], dibutyryl-cAMP [#D0627, Sigma-Aldrich], TNF-α [#T7539, Sigma-Aldrich] or insulin [#I9278, Sigma-Aldrich] in combination with 8-Bromoadenosine 3*'*,5*'*-cyclic monophosphate sodium salt [#B7880, Sigma-Aldrich] for 3 h at 37°C. Glycerol release into the culture media was subsequently quantified to determine basal and stimulated lipolysis as described previously [26].

### Lipogenesis assay

Cells were cultured as described above with the following modifications: **i)** at day 11 of differentiation, insulin was removed from the media, **ii)** at day 13, cells were incubated in DMEM without glucose [#F0405 Biochrom AG, Merk] and **iii)** at day 14, cells were incubated in glucose-free DMEM supplemented with 1 μM glucose for 3 h [#A24940, Thermo Fisher Scientific]. Subsequently, cells were culture in glucose-free DMEM supplemented with 0.125 mCi [3-^3^H]-D-glucose [#NET331A001MC, PerkinElmer] and 1 µM glucose DMEM with or without insulin [#I9278, Sigma-Aldrich] for 3 h. Prior to lysis in H_2_O supplemented with 0.1% SDS [#I4509, Sigma-Aldrich], cells were washed three times in cold PBS. One part of the lysate was used to determine protein concentration using the BCA Protein determination kit and the other part the lysate was added to scintillation liquid (Optiphase HiSafe 3 scint solution [#1200.437, PerkinElmer]) and counting per minute (CPM) was measured using a β-counter [Trib Carb 4910 TR, PerkinElmer] using the QuantaSmart software.

### Proteomics

#### Proteomics sample preparation

Samples for proteome measurement were prepared using the inStageTip (iST) method [27]. In detail, frozen samples were mixed with SDC buffer (100 mM Tris-HCl, pH 7.6, and 2% (w/v) SDC) to a final concentration of 1% SDC, heated (5 min, 95°C) and sonicated (Diagenode Bioruptor, high intensity, 15 × 30 s). After determining protein concentration by BCA assay, 30 µg protein were reduced and alkylated using 10 mM TCEP and 40 mM CAA (5 min, 40°C in the dark). Samples were digested with trypsin and LysC (1:50 protein:enzyme) overnight at 37°C. Peptides were acidified with TFA (1% final concentration) and loaded onto activated triple layer styrenedivinylbenzene – reversed phase sulphonated STAGE tips (SDB-RPS; 3 M Empore) and washed with 100 µl ethylacetate 1% TFA, 100 µl 30% methanol 1% TFA and 150 µl 0.2% TFA. Peptides were eluted with 60 µl SDB-RPS elution buffer (80% ACN, 5% NH_4_OH), followed by evaporation of the solution in a SpeedVac for 40 min at 45°C. Desalted peptides were re-solved in 10 µl MS loading buffer (2% ACN, 0.1% TFA) and stored at −20°C until measurement.

#### LC-MS/MS analysis

Proteomes were measured using a 115-min long single-shot data-independent acquisition (DIA) method on an Orbitrap Exploris 480 mass spectrometer [Thermo Fisher Scientific]. For this, 500 ng of peptide mixture were separated on an in-house packed column (1.9 µm C18 ReproSil particles, Dr. Maisch GmbH) using the EASY-nLC 1200 liquid chromatography unit [Thermo Fisher Scientific]. Peptides were separated on a binary buffer system consisting of buffer A and B (0.1% formic acid and 80% ACN, 0.1% formic acid, respectively) in which buffer B changed from 5% to 45% over the elution. Peptides were ionized via electrospray (ESI) and pre-filter b FAIMS [Thermo Fisher Scientific] switching between a constant voltage (CV) of −50 V/-70 V each corresponding to a full ms1/ms2 DIA cycle. The cycle consisted of a ms1 scan (300–1650 m/z, max. ion fill time of 45 ms, normalized AGC target = 300%, *R* = 120.000 at 200 m/z) followed by 49 tMS2 fragment scans of unequally spaced windows in the same m/z range (fill time = 22 ms, normalized AGC target = 1000%, normalized HCD collision energy = 30%, *R* = 15.000).

#### Bioinformatic workflow and data analysis

The DIA raw files were processed using Spectronauts (v14.9.201124.47784) directDIA function [28] with default settings. Spectra were searched against a human UniProt regular and “additional” FASTA database (UP000005640_9606_2020–06; 20609 and 77,157 entries respectively) and filtered for contaminants (MaxQuant contaminants list, 245 entries). Spectronaut report files were further analysed with Perseus (v.1.6.14.0).

### Transcriptomics

Total RNA from hAPCs (P12) and TERT-hAPCs (P52) was subjected to quality control with Tapestation (Agilent) according to the manufacturer’s instructions. To construct libraries suitable for Illumina sequencing, the stranded mRNA sample preparation protocol was used with starting concentration between 25 and 1000 ng total RNA. The protocol includes mRNA isolation, cDNA synthesis, ligation of adapters and amplification of indexed libraries. Library yield and quality were analysed using a Qubit (Thermo Fisher Scientific) and a Tapestation (Agilent), respectively. The indexed cDNA libraries were normalized and combined, and the pools were sequenced on the Illumina Nextseq 2000 P2 100 cycle sequencing run, generating 58 base paired end reads with dual index. Basecalling and demultiplexing was performed using Illumina bcl2fastq (v2.20). Sequence data quality was assessed using FastQC (v0.11.8). Reads were aligned to Ensembl GRCh38 reference genome using STAR (v2.6.1d). Gene counts were estimated using featureCounts (v1.5.1). Count data was imported to Bioconductor package limma-Voom (v3.53.1) and tested for differential expression between different cell type. Gene set over-representation analysis for the significantly regulated genes at different comparison was performed using Enrichr function in ClusterProfiler package (v 4.4.2). The Hallmark gene set from MsigDB (Broad Institute) was used to calculate the enrichment.

### Integration of proteomic and transcriptomic data

The transcriptomic and proteomic data were analysed as described under the respective header. The resulting tables from these analyses were overlapped based on gene symbols and 4,700 genes were found to be present in both data sets. Out of these, 588 entries displayed congruent mRNA and protein regulation at a false discovery rate < 0.05 comparing edited vs. non-edited adipocytes.

### Imaging of 2D cell cultures

Cells were plated in 170-μm-thick glass-bottom 96 or 24 well plates [#5242–20 or #5232–20, zell-kontakt] and cultured as described above. On day 13 of differentiation, cells were fixed in PBS supplemented with 4% paraformaldehyde [#SC281692, Santa Cruz Biotechnology] for 10 min at room temperature and washed three times with PBS. Lipid droplets and nuclei were stained by incubating the cells for 10 min at room temperature with PBS supplemented with BODIPY 493/503 [1:2500, #D3922, Thermo Fisher Scientific] and Hoechst 33,342 [1:5000, #H21492, Invitrogen], respectively. Subsequently, cells were washed three times with PBS. Images were acquired using a Nikon Spinning disk CREST v3 inverted microscope with BSIexpress sCMOS camera (6.5um pixels) and using 20×/0.75 air and 60×/1.2 water objectives.

### Imaging of 3D cell cultures

Spheroids were cultured as described above, fixed for 1 h in PBS supplemented with 4% paraformaldehyde and stained with BODIPY 493/503 and DRAQ5 [1:1000, #65088092, Thermo Fisher Scientific]. Spheroids were mounted in 1.2% low melting point agarose in 170-μm-thick glass-bottom 24 well plates [#5232–20, zell-kontakt] and cleared with the SeeBD method as previously described [4]. Z-stacks were acquired using a Nikon Spinning disk CREST v3 inverted microscope with BSIexpress sCMOS camera using a 25×/1.05 silicone objective and denoised using the NIS elements Denoise.Ai pre-trained algorithm. Representative images are displayed as maximum intensity projections.

### Statistics and data availability

Upon acceptance of this work or reviewers’ requests, transcriptomic and proteomic raw data will be available through the Gene Expression Omnibus repository and the Proteomics Identification Database, respectively. Results are expressed as mean ± SEM and statistical tests are indicated in the figure legends. Data analyses were performed using GraphPad Prism software (version 9.0, San Diego, CA) and statistical significance is indicated as: *p < 0.05, **p < 0.01, ***p < 0.001, ****p < 0.0001.

## Supplementary Material

Supplemental Material

Figure S1 R1 for submission.jpg

Figure S2 R1 for submission.jpg

## Data Availability

The data that support the findings of this study are available on request from the corresponding author; MR. The data are not publicly available due to their containing information that could compromise the privacy of research participants.
