# Supplementary figures and images for: Adipogenic characterization of immortalized CD55+ progenitor cells from human white adipose tissue
Source: Adipocyte. 2023 Nov 20;14(1):2283213. doi: 10.1080/21623945.2023.2283213 (PMC12147497; doi:10.1080/21623945.2023.2283213)

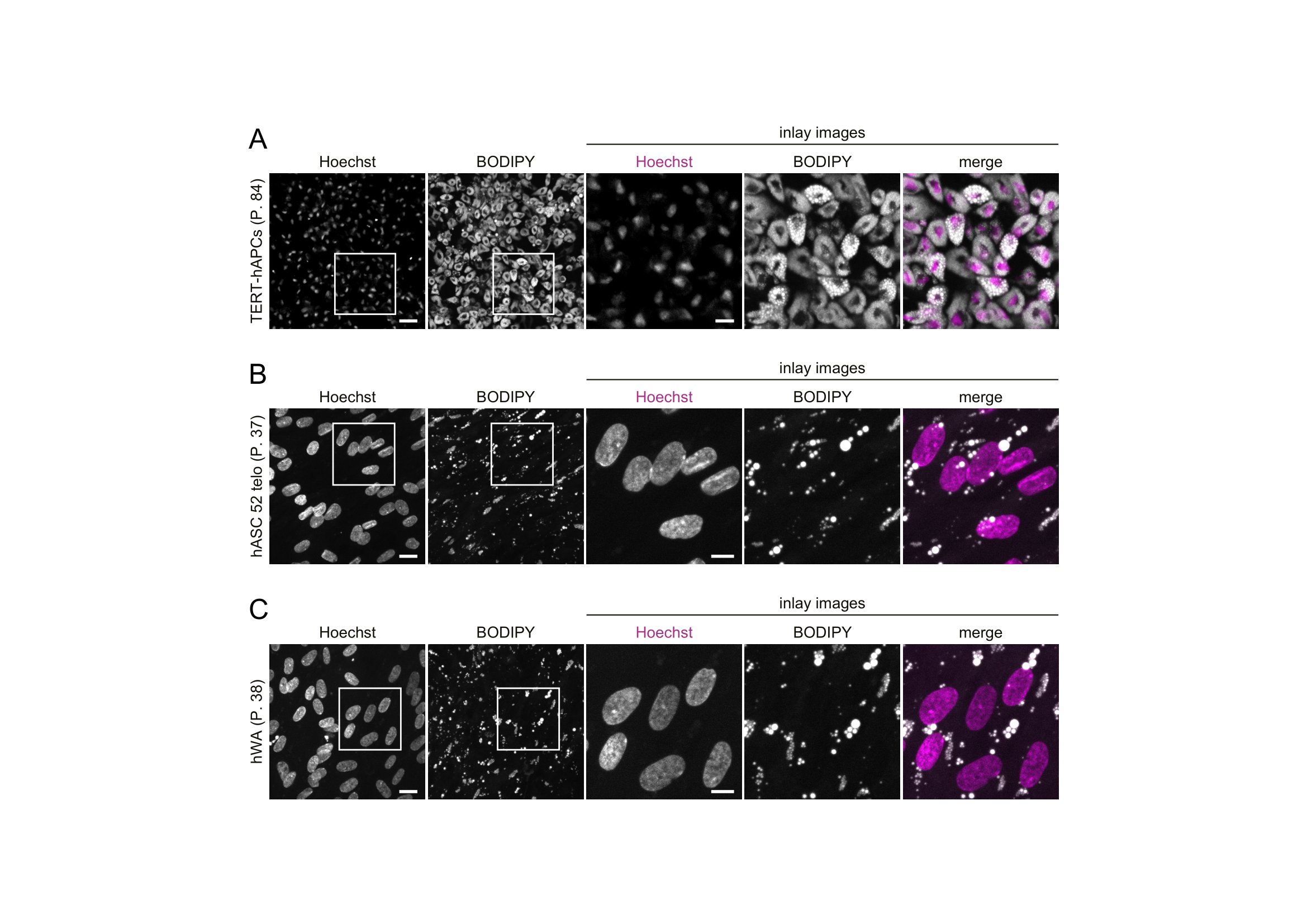

Supplement: Figure S1 R1 for submission.jpg [file KADI_A_2283213_SM6507.jpg]

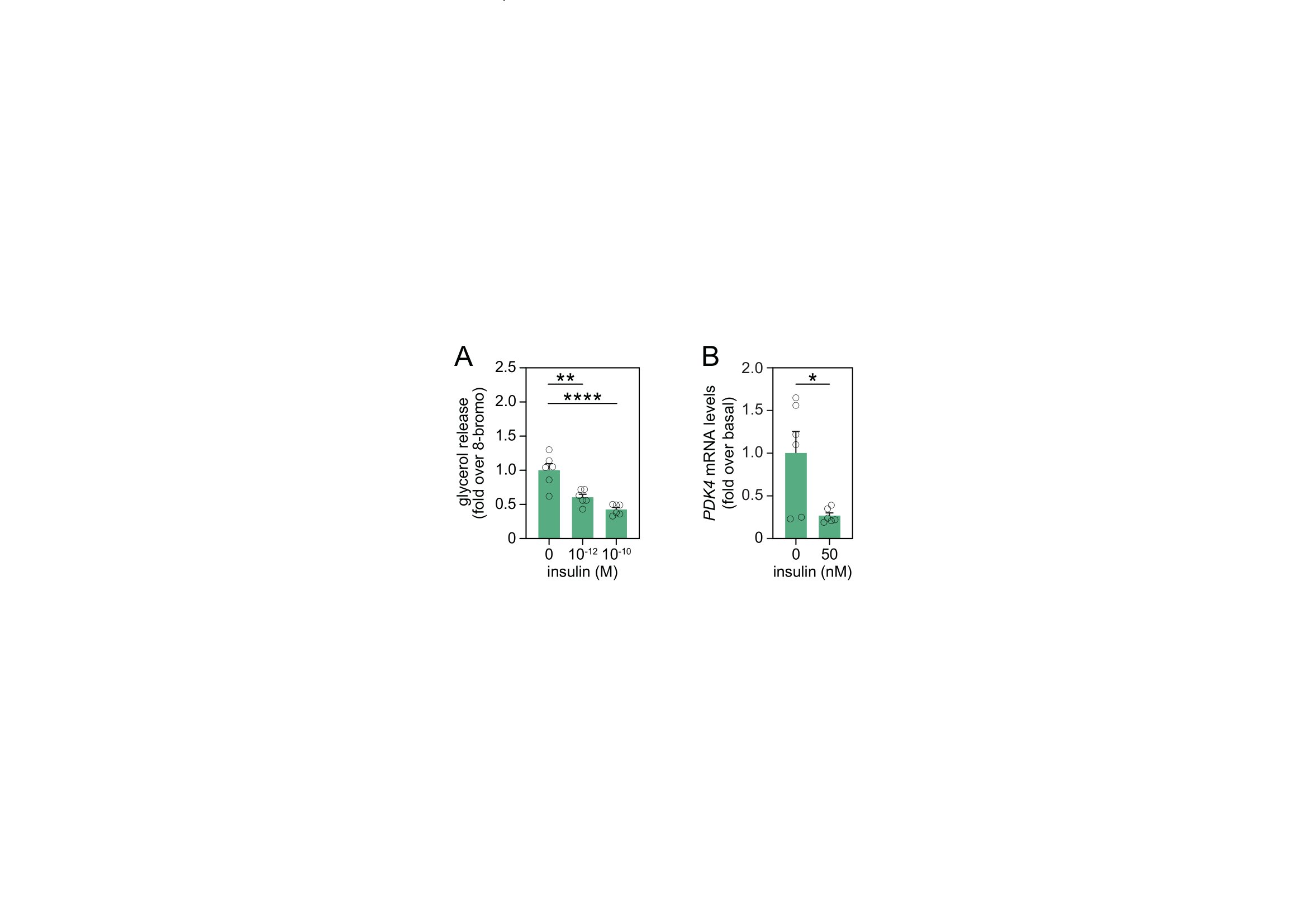

Supplement: Figure S2 R1 for submission.jpg [file KADI_A_2283213_SM6506.jpg]
